# Assessment of novel Lehmann’s funnel entry trap prototypes performance to control malaria mosquito populations

**DOI:** 10.1186/s12936-020-03532-x

**Published:** 2021-01-01

**Authors:** Roger Sanou, Hamidou Maïga, Etienne M. Bilgo, Simon P. Sawadogo, Bazoumana B. D. Sow, Adama Ouema, Koama Bayili, Adrien Marie Gaston Belem, Léa Paré Toé, Roch K. Dabiré, Abdoulaye Diabaté

**Affiliations:** 1 Institut de Recherche en Sciences de La Santé (IRSS)/Centre Muraz, Bobo-Dioulasso, Burkina Faso; 2 Université Nazi BONI de Bobo-Dioulasso, PO 1091, Bobo-Dioulasso, Burkina Faso

**Keywords:** Prototypes, Adult mosquito trap, *Anopheles gambiae*, Malaria, Burkina faso

## Abstract

**Background:**

There is a global consensus that new intervention tools are needed for the final steps toward malaria elimination/eradication. In a recent study in Burkina Faso, the Lehmann Funnel Entry Trap (LFET) has shown promising results in the reduction of mosquito densities, even in areas where insecticide resistance is as high as 80%. The LFET requires no chemicals and is self-operated. However, one of the issues with the original LFET is the size of the funnel, which often occupies too much space within users’ homes. Here, the performance of three new, smaller-sized LFET prototypes that combine a screening and killing effect on mosquitoes was assessed.

**Methods:**

The study was carried out over three months during the rainy season in low and high malaria vector density sites, Soumousso and Vallée du Kou, respectively. The original LFET (or ‘Prototype 1’/‘P1’) was modified to produce three new prototypes, which were referred to as prototype 2 (‘the Medium’ or ‘P2’), prototype 3 (P3) and prototype 4 (P4). Each of the new prototypes was tested on eight days per month over the three-month period to assess their effectiveness in trapping and killing mosquitoes entering houses through the windows compared to the original LFET.

**Results:**

Overall, 78,435 mosquitoes (mainly *Anopheles gambiae *sensu lato) were collected in the two study sites, both in the traps and in the houses. A total of 56,430 (72%) mosquitoes were collected from the traps. In Vallée du Kou, the original LFET caught a greater number of mosquitoes than the medium (prototype 2), whereas no difference was observed between the other new prototypes (3 and 4) and the medium. In Soumousso, both the original and medium LFETs collected significantly greater numbers of mosquitoes compared to prototypes 3 and 4.

**Conclusion:**

This study has shown that the new LFET prototypes are effective in trapping mosquitoes in high mosquito density settings. A large-scale study with one of the prototypes will be needed to assess community acceptance of the traps and their ability to control malaria vectors.

## Background

Malaria has decreased dramatically over the last decade, and the number of deaths has dropped from 445,000 in 2016 to 405,000 in 2018 globally [1, 2], mainly due to the up-scaling of vector-control interventions. Current vector control relies primarily on the use of long-lasting insecticidal nets (LLINs) and indoor residual spraying (IRS) [3]. However, the spread of insecticide resistance across *Anopheles* mosquito species is threatening and undermining the global effort for malaria elimination [1, 4]. In order to manage the spread of mosquito insecticide resistance, one important consideration is the effective monitoring of mosquito vector populations, a key element of vector management and assessment of mosquito borne disease [5]. Therefore, there is an urgent need to identify novel tools for malaria control.

Malaria transmission is mediated by female *Anopheles* mosquitoes. Female mosquitoes seek blood meals late at night in human dwellings [6–8] when people are vulnerable. Although malaria is mostly transmitted indoors, some studies in East and West Africa have shown malaria transmission occurring outdoors by mosquitoes that escape insecticides or other indoor control methods [9–12]. Mosquito entry rate and consequently disease transmission are affected by house type [13], and it is widely acknowledged that poor quality housing—constructions with wooden roofs, straw, clay bricks and roof plates, or clay roofs, for example—is generally believed to be an important contributor to ill health [14]. Recently, new approaches based on the design of house ceilings, doors, windows and eaves were developed to reduce mosquitoes entering into houses and thus transmitting disease [15, 16]. Furthermore, approaches that exploit insect behaviour with regards to house entry have been explored as part of malaria vector control strategies [17, 18]. These ideas were brought together in the design of the original Lehmann’s Funnel Entry Trap (LFET), a window trap exploiting mosquito endophilic and anthropophilic behaviours and entry route [19]. The potential of the original LFET to control mosquito densities has been demonstrated, and preliminary results showed house entry reduction by 71% in Vallée du Kou, a high mosquito density area in Burkina Faso [19]. However, the original LFET prototype occupied too much space, impacting residents’ enthusiasm for continuous use. In response to this major issue, the original LFET was scaled down.

Here three smaller LFET prototypes’ trapping efficiency was evaluated and compared to the original LEFT [19] in low and high mosquito density areas. In addition, this study aimed to determine a promising prototype that could be proposed/recommended as a vector control tool.

## Methods

### Study areas

**Fig. 1:**
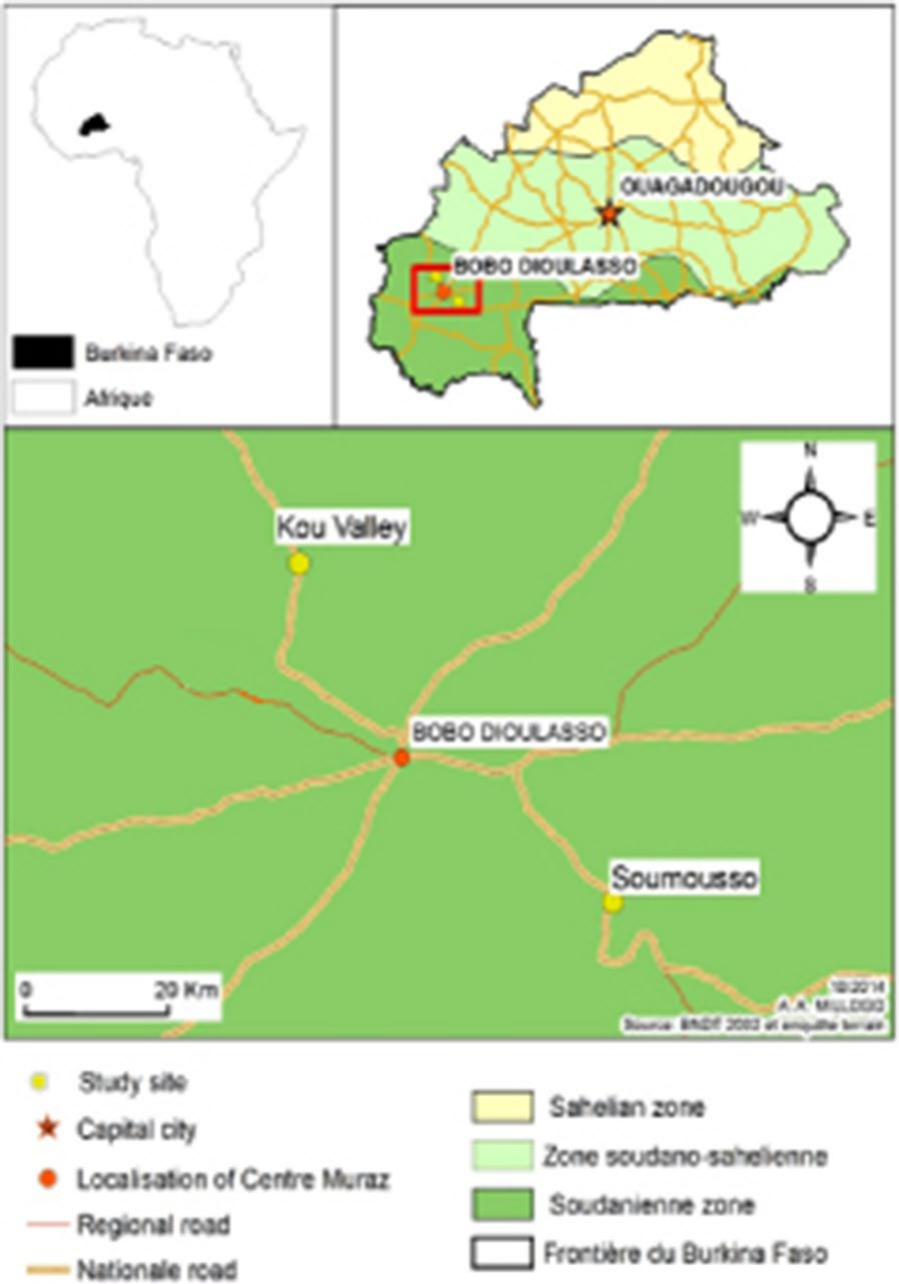
Map of the study sites, Vallée du Kou (Kou Valley) and Soumousso

The study was carried out in two ecological settings, Vallée du Kou and Soumousso (Fig. 1), located near Bobo-Dioulasso, Burkina Faso. Vallée du Kou (11°23′ N, 4°24′ W) is a village located to the north-west of Bobo-Dioulasso, characterized by over 1200 ha of wooded savannah. It is a rice growing area with high mosquito densities throughout the year. The trial was conducted in one village of the seven neighbourhoods separated from one another by rice fields, Vallée du Kou 3 (‘VK3’). This site was chosen due to its proximity to the main tarred road. Given the presence of surface water all year round, mosquitoes are found with ease, with a peak density observed in August–September, during the rainy season. *Anopheles gambiae* and *Anopheles coluzzii* are present with a predominance of *An. coluzzii* throughout the year. Both species in this area are highly resistant to pyrethroids and DDT (*kdr* based mechanism, 0.8–0.95), and with an increase in *ace-1* resistance frequency has also been reported [20, 21].

**Soumousso** (11°04′ N, 4°03′ W), located in the south of Bobo-Dioulasso, in contrast with VK3, it is a drier setting where the dominant species are *An. gambiae,* and a mixture of mostly *Anopheles funestus*, *Anopheles arabiensis, Anopheles coluzzii* [22]. In this area, the mosquito density is lower compared to that of Vallée du Kou, and the dynamics of the mosquito population follow the two main seasons, with fewer mosquitoes in the dry season compared to the rainy season.

### Description of the traps

**Fig. 2:**
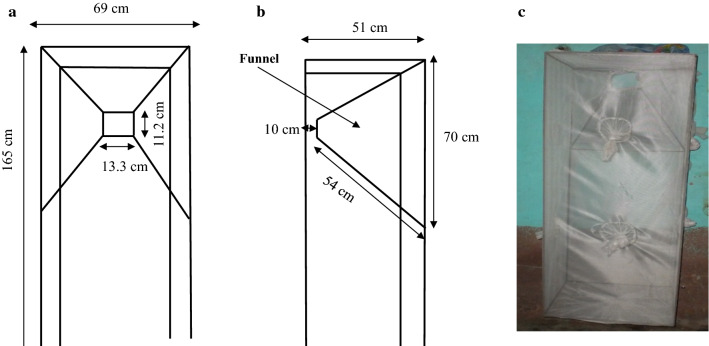
Dimensions of the trap, front and side view. **a** Inserts front view: 69 cm wide × 165 cm high; 13.3 cm long × 11.2 cm wide (small opening of the funnel). **b** Inserts side view: 51 cm depth of the trap, 70 cm long × 54 cm diagonal (large opening of the funnel); 10 cm distance of the small opening of the funnel from the backside of the trap (Diabaté et al. [19]); **c** original prototype outside view inside a house

The original LFET (prototype 1, P1) was designed by Diabaté and collaborators in 2013 [19] and was made from a metal frame (length = 69 cm, width = 51 cm, height = 165 cm) fitted with a regular mosquito net to prevent mosquitoes and other insects from escaping the trap once they enter it (Fig. 2). A funnel made from a metal frame was inserted at the top of the trap in such a way that mosquitoes approaching the window go first through the larger opening of the funnel and enter the trap through the small and rectangular opening. The first (large) opening of the funnel is 70 cm long and 54 cm diagonally, while the second (small) opening in bottom 13.3 cm long and large of 11.2 cm. The small opening of the funnel is 10 cm away from the backside of the trap. The funnel is inserted in the frame in a way that allows the mosquitoes to enter the trap easily but prevents them from escaping. Once the mosquitoes enter the trap, they have a large space beneath the funnel where they disperse. For a mosquito to escape, it would have to fly up towards the small opening of the funnel and navigate through the 10 cm space separating the small opening of the funnel and the back of the frame. Ultimately, mosquitoes continue to fly to exhaustion before finding a way out. The principle of the LFET is to confine the mosquito inside the trap until dehydration and death. For the purpose of this experiment, traps were fitted with three sleeves on the side (one below, one in the middle and one on the top) through which mosquitoes were aspirated. The trap was secured to the windows using nails.

**Fig. 3:**
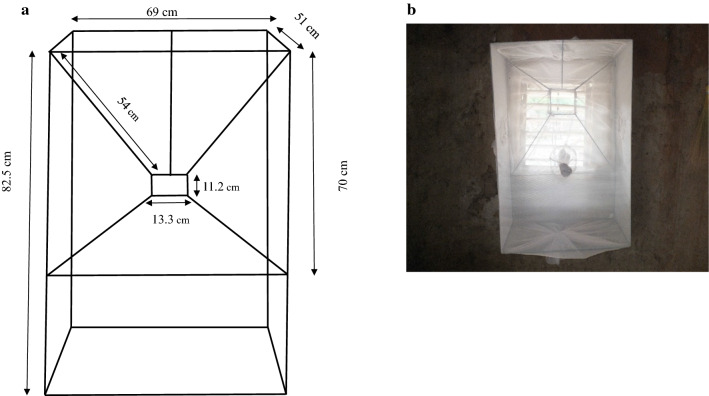
**a** Dimensions of the trap, front and side view. Inserts front view: 69 cm wide × 82.5 cm high; 13.3 cm long × 11.2 cm wide (small opening of the funnel). Inserts side view: 51 cm depth of the trap, 70 cm long × 54 cm diagonal (large opening of the funnel); 10 cm distance of the small opening of the funnel from the backside of the trap. **b** Prototype 2 outside view inside a house

The medium (prototype 2, P2) is a smaller version of the original LFET. The funnel dimensions are similar but its height (82.5 cm) is half that of the original LFET (165 cm) (Fig. 3). Similarly, the whole trap was also covered with a net, fitted with three sleeves for mosquito collection.

**Fig. 4:**
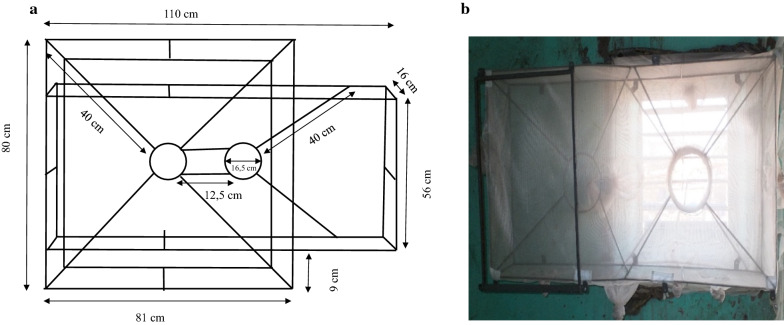
**a** Dimensions of the trap, inserts front view. 16 cm wide × 80 cm high; 16.5 cm diameter (circular opening of the funnel), 56 cm depth of the trap, 81 cm long × 40 cm diagonal (large opening of the funnel); 10 cm distance of the small opening of the funnel from the backside of the trap. **b** Prototype 3 outside view inside a house, with circular funnel as entrance

The Prototype 3 (P3) was made using a metal funnel frame with the following measurements: length = 81 cm, width = 16 cm, height = 80 cm (Fig. 4). Here, the small funnel opening used as entry is a circular metal funnel, instead of rectangular as in the previous traps, with an opening size of 16.5 cm in diameter. Two circular openings on the right and on the left (the distance between both circular openings is 12.5 cm) were made to fit the window size. Depending on the space available around the window of the house, the trap position may allow the use of only one of the openings (either right or left). When one opening is used as entry, the second would be closed and covered with netting. The volume of the trap was made of a horizontal and rectangular metal frame (length = 110 cm, width = 16 cm, height = 56 cm). A net covered the trap on the backside of the funnel and was fitted with five sleeves allowing mosquito collection.

The prototype 4 (P4) is similar to P3 in terms of size and funnel type but has an additional circular funnel frame. This small (9 cm long) circular funnel gives access to the space beneath.

**Fig. 5:**
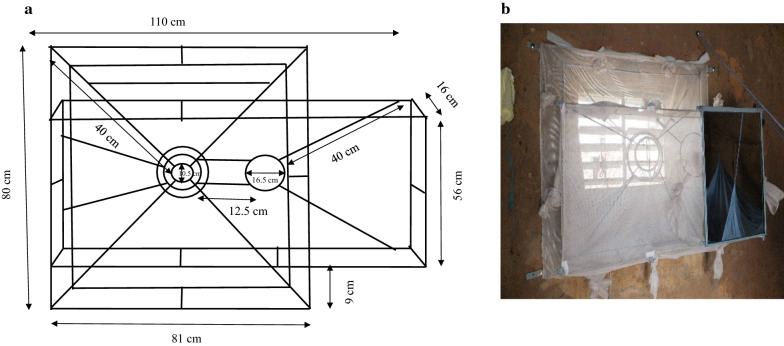
**a** Dimensions of the trap, inserts front view. 16 cm wide × 80 cm high; 16.5 cm diameter (circular opening of the funnel), 56 cm depth of the trap, 81 cm long × 40 cm diagonal (large opening of the funnel); small circular funnel diameter 10.5 cm, distance from the beginning of the large circular to the end of small circular 9 cm and 10 cm distance of the small opening of the funnel from the backside of the trap. **b** Prototype 4 outside view inside a house, with small circular funnel entrance

The distance between the end of the circular pipe and the back of the trap is 10 cm. In addition, P4 was equipped with a mirror for the inhabitants’ personal use, which would make it valuable for other functions beyond controlling mosquito populations. This may help increase the acceptability and sustained use of the trap (Fig. 5). As with P3, this trap was also outfitted with five sleeves enabling mosquito collection.

### Study design and mosquito collection

**Fig. 6:**
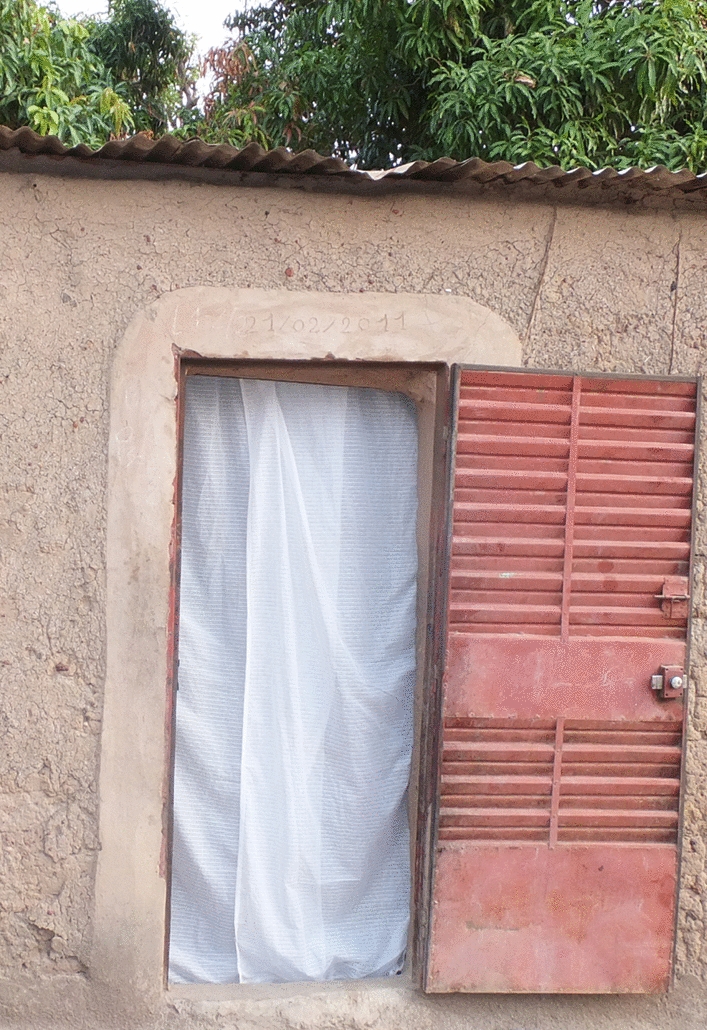
A curtain placed at the door of a study house

Three of each of the new LFET prototypes (medium/P2), P3 and P4) were tested in the two selected ecological settings along with three of the original LFET design (P1) for comparison purposes. The performance of the traps was assessed in terms of the number of malaria mosquitoes trapped as well as other mosquitoes entering the house through the window. A total of 12 houses, corresponding to 12 traps, were chosen in each site (a total number of 24 traps produced for both sites). Only houses with a single room, single window (similar size with a metal frame) and single door were selected for the study. Each of the houses were at least 10 m apart from each other. All of the traps were installed on the same day between 15:00 and 17:00 with a two-day rotation between houses according to a Latin square plan, to reduce biases linked to house inhabitants’ attraction. After installation, all the traps were simultaneously used, and checked every morning for eight consecutive days per month, for collection of all mosquitoes dead or alive in the traps for morphological identification. To ensure that mosquitoes had no other alternative except the windows to enter the house, small holes in ceilings and walls were blocked using sponges or cloth, and a curtain was placed at the entrance of each house (Fig. 6). The inhabitants were informed of the aim of the study and, therefore, free to use their doors as they wished. The window where the LFET was fitted was left open throughout to allow mosquitoes to enter the house. Traps were installed the day before prior to mosquito sampling over eight consecutive days per month, from September to November in VK3 and Soumousso.

Traps were emptied from 07:00 to 09:00, using a mouth aspirator in addition to pyrethrum spray catches [23] performed in the corresponding house. All the mosquitoes caught were kept in a single cup, then killed with chloroform and morphologically identified on site. The daily work on site consisted of mosquito collection, sorting, identification, sexing according to Gillies and De Meillon [24], counting and scoring per genus, species and physiological status (unfed, blood fed, gravid), and the numbers were recorded on a spreadsheet. All traps set up each month were removed at the end of the eight-day period.

### Statistical analysis

Microsoft Excel 2007 (Microsoft^®^, New York, USA) was used to record the data, and R-3.6.2 (package dplyr, questionr, and coin) was used for statistical analyses and to produce the graphs.

In the study, three main variables were analysed: (1) T = Tt + Th, T = Total number of *An. gambiae *sensu lato (s.l.) collected in the trap and in the house, Tt = total number of mosquitoes collected in trap, Th = total number of mosquitoes collected in the house; (2) P (%) = (Tt/T)*100, P = Proportion (%) of mosquito entry reduction in the house and (3) Dr = T/24, Dr = daily removal of mosquitoes per site over 24 days.

The number of mosquitoes in the traps and the matching houses did not follow a normal distribution. Therefore, a one-way non-parametric analysis of variance (Kruskal Wallis test) was used to determine whether there was difference between the traps in terms of numbers of mosquitoes collected and to assess their overall performance.

The post-hoc test was used for multiple comparisons of mean numbers of mosquitoes between traps (Bonferroni). In order to meet this post-hoc test assumption, a Tukey test for multiple comparisons was used to confirm the results.

The effects of monthly collection in mosquito density reduction were evaluated using a Levene’s test for homogeneity of variance (centre = median) with a Wilcoxon rank sum test and a p value bonferroni adjustment method. A Tukey multiple comparison of means with 95% family-wise confidence level test was used for pair-wise comparison between LFET prototypes.

To assess whether the traps caught more mosquitoes than those that entered in the matching houses, a comparison using Wilcoxon rank sum test with a holm p-value adjustment method was used.

## Results

### Mosquito density reduction

Overall, 78,435 mosquitoes were collected in the two study sites and were composed of 76,558 (98%) *An. gambiae* s.l. (Table 1) and 1,877 (2%) other species, which included *An. funestus, Anopheles coustani, Anopheles flavicosta, Anopheles pharoensis, Anopheles rufipes, Mansonia* sp, *Culex* sp and *Aedes* sp (Table 2). Out of 76,558 *An. gambiae* mosquitoes collected in both traps and houses, 75,471 were caught in VK3 and 1087 in Soumousso, whereby 72% and 60% respectively were collected from the traps (Table 1).

**Table 1:** Total and proportion of male and female *Anopheles gambiae* s.l. mosquitoes collected per trap vs house per village

Village/type of trap	VK3		Total (trap + house)	Soumousso		Total (trap + house)	Trap (%) in both sites
	Trap, % (n)	House, % (n)		Trap, % (n)	House, % (n)		
Original	78.64 (18,230)	21.35 (4950)	23,180	73.27 (233)	26.73 (85)	318	78.6
Medium	70.21 (10,130)	29.79 (4298)	14,428	72.80 (289)	27.20 (108)	397	70.3
Prototype 3	69.53 (13,135)	30.47 (5755)	18,890	35.90 (56)	64.10 (100)	156	69.3
Prototype 4	69.04 (13,099)	30.96 (5874)	18,973	38.89 (84)	61.11 (132)	216	68.7
Total	72.33 (54,594)	27.66 (20,877)	75,471	60.90 (662)	39.10 (425)	1087	72.2

n = number of mosquitoes per trap or in house, (%) = proportion of (n) mosquitoes in trap or in house/total (trap + house)

**Table 2:** Numbers of other mosquito species caught in trap versus house in both sites over the study period

Species	VK3						Soumousso					
	Original	Medium	P3	P4	Total species/trap	House	Original	Medium	P3	P4	Total species/trap	House
*Culex* spp.	369	275	183	142	969	538	42	33	15	16	106	153
*An. pharoensis*	8	9	7	5	29	3	1	0	2	0	3	0
*Mansonia* spp.	6	12	5	5	28	3	0	0	0	0	0	0
*An. coustani*	5	8	7	3	23	3	0	1	0	0	1	0
*An. rufipes*	0	0	0	0	0	0	1	0	0	1	2	2
*An. funestus*	0	0	0	0	0	0	3	2	0	0	5	0
*An. flavicosta* *Aedes*. spp	0 0	0 0	0 0	0 0	0 0	0 0	1 0	6 1	0 0	0 0	7 1	0 1
Total	388	304	202	155	1,049	547	48	43	17	17	125	156

P = LFET prototype

**Fig. 7:**
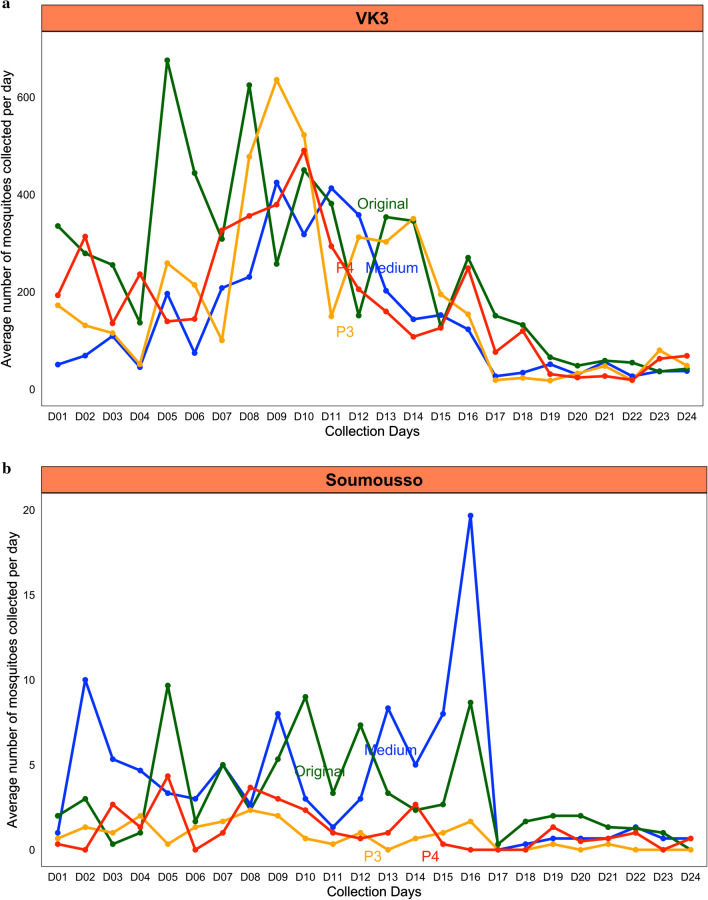
Mean number of mosquitoes collected per Lehmann’s Funnel Entry Trap prototype/day. **a** In VK3 and **b** in Soumousso

In VK3, the original LFET (P1) collected a daily average number of mosquitoes ranged from (36 to 675) in November (the end of the rainy season) and September (mid rainy season) respectively, while the medium LFET (P2) collected (26 to 414) and prototypes 3 and 4 collected (17 to 635) and (19 to 490), respectively (Fig. 7a).

In Soumousso, the daily average number of mosquitoes collected were (0–9, 0–19, 0–2) and (0–4) mosquitoes per trap during the trapping period for original, medium, P3 and P4, respectively (Fig. 7b). The new traps (P3 and P4) performed better in terms of house entry reduction in high mosquito density VK3, with 69% of mosquitoes denied access to the houses as compared to (36% for P3 and 39% for P4) in the low-density site, Soumousso. In addition, during the study in VK3, the original LFET reduced house entry by 78%, as compared to 70% for the medium LFET. The original LFET (P1) reduced mosquito entry by 73% while the new prototypes (medium (P2), P3, and P4) reduced house entry by a range of 36% (P3) to 73% (medium) in Soumousso (Table 1).

**Fig. 8:**
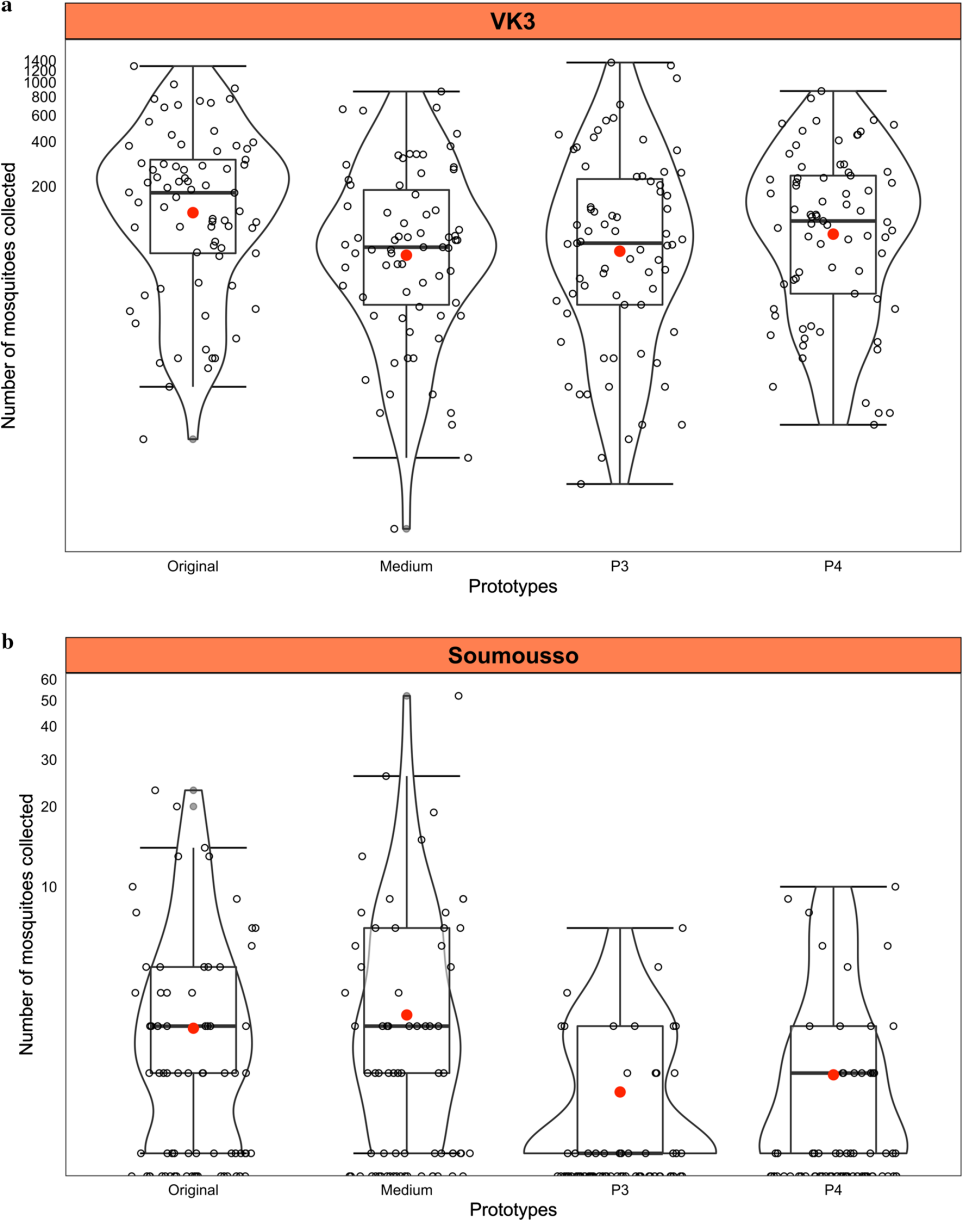
Number of mosquitoes collected per trap prototype **a** in VK3 and **b** in Soumousso

The average daily *An. gambiae* mosquito removal (Dr) was 262/house/night/trap in VK3 and 3.77/house/night/trap in Soumousso during the study period, (Fig. 8) summarizes the variance of the number of mosquitoes collected per trap/month/site.

The seasonal effect of month on the mosquito collection in traps showed a significant variation according to the study site (Kruskal–wallis, χ^2^ = 10.9, df = 3, p = 0.012 in VK3; and χ^2^ = 40.7, df = 3, p < 0.0001 in Soumousso). The variation level in performance between the traps was confirmed, by Levene’s test for homogeneity of variance (centre = median) (group 284, df = 3, F value = 1.9, Pr (> F) = 0.13 in VK3) and (group 283, df = 3, F value = 6.56 Pr (> F) < 0.001 in Soumousso).

**Fig. 9:**
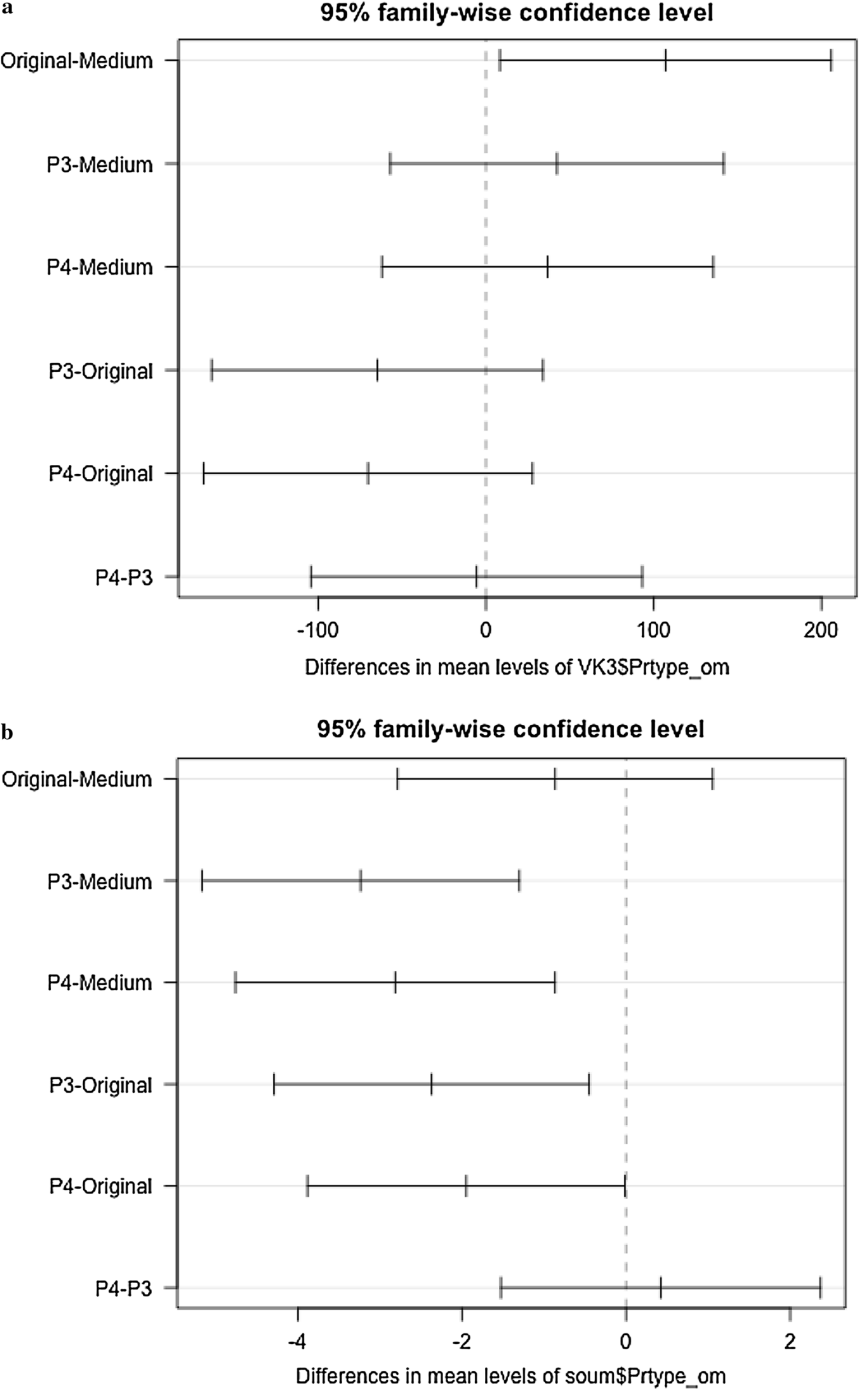
Tukey multiple comparisons of means 95% family-wise confident level **a** in VK3 and **b** in Soumousso

The original LFET caught a greater number of male and female *An. gambiae* mosquitoes than the medium (P2) (p = 0.014), whereas no difference was observed between the medium and other new prototypes (P3 and P4) in VK3 (Fig. 9a).

In Soumousso, the original (P1) and medium (P2) traps collected a similar number of male and female *An. gambiae*, but significantly higher than that of Prototypes 3 and 4 (p < 0.0001) (Fig. 9b). A greater number of mosquitoes were caught in the traps compared to the matching houses (p ˂ 0.0001) (Additional file 1: Fig. 10).

### Gonotrophic status of collected mosquitoes

Out of 76,558 *An. gambiae* caught in both traps and houses, 58,439 (76%) were females and 18,119 (24%) were males. Of the females, 42,116 (72%) were caught in the traps whereas 16,323 (28%) were collected in the houses. Of the 42,116 trapped mosquitoes, 18,227 were unfed and likely seeking a blood meal represented (43%), while 19,994 (48%) were blood fed and 3895 (9%) were gravid females (Table 3).

**Table 3:** Gonotrophic status of female mosquitoes and number of males caught in trap versus house in both sites over the study period

Village	Prototype or house	Unfed, % (N)	Bloodfed, % (N)	Gravid, % (N)	Male, % (N)	Total
VK3	Original	39.27 (7159)	30.69 (5594)	6.9 (1258)	23.14 (4219)	18,230
VK3	Medium	37.58 (3837)	32.79 (3322)	7.01 (711)	22.31 (2260)	10,130
VK3	Prototype 3	27.42 (3602)	37.39 (4911)	5.89 (774)	29.30 (3848)	13,135
VK3	Prototype 4	24.03 (3148)	46.27 (6061)	8.48 (1111)	21.22 (2779)	13,099
Soumousso	Original	75.11 (175)	15.02 (35)	5.58 (13)	4.29 (10)	233
Soumousso	Medium	77.51 (224)	12.46 (36)	5.88 (17)	4.15 (12)	289
Soumousso	Prototype 3	75.00 (42)	16.07 (9)	5.36 (3)	3.57 (2)	56
Soumousso	Prototype 4	47.62 (40)	30.95 (26)	9.52 (8)	11.90 (10)	84
VK3	House	23.05 (4813)	38.64 (8067)	14.57 (3042)	23.73 (4955)	20,877
Soumousso	House	2.59 (11)	70.12 (298)	21.65 (92)	5.65 (24)	425
Total (VK3 + Sounousso)	Trap + house	30.11 (23,051)	37.04 (28,359)	9.18 (7029)	23.67 (18,119)	76,558

NB: total in house = number of mosquitoes collected in all houses over the study period

## Discussion

The objective of this study was to assess the modified LFET prototypes’ performance in terms of suppressing malaria vectors, and to determine the most promising prototype as a vector control tool. The new LFET designs have shown the potential to reduce malaria mosquito densities in VK3. Conversely, in Soumousso, aside from the medium (P2), the new prototypes showed a relative low-density mosquito reduction in the houses compared to the original (P1). In addition to *An. gambiae*, other mosquito species responsible for neglected tropical diseases such as lymphatic filariasis and dengue were collected.

These results are consistent with the previous study [19] where the original design (P1) was able to reduce the number of mosquitoes from 70 to 80% in houses in a high mosquito density area. This shows that the size of the new prototypes did not impact on their performance. In addition to the smaller size (reducing the cost of manufacturing) of the modified LFET, Prototype 4 has a mirror that can be used by residents, making the trap design more attractive and acceptable to potential users. The new traps cost ~ $35, ~ $40 and ~ $41 (USD) for the medium (P2), prototype 3, and prototype 4, respectively. This compares to $42 for the original LFET (Table 4).

**Table 4:** Cost of manufacturing and length of metal used for the trap prototypes

Trap design	Length of metal used (m)	Cost of manufacturing (US$)
Original	~ 15.81	~ 42
Medium	~ 12.51	~ 35
Prototype 3	~ 13.23	~ 40
Prototype 4	~ 13.58	~ 41

The new prototypes were able to block the entry of malaria mosquitoes and other insects into the house, and thus prevent the harmful effects of mosquito bites. The design of the medium LFET was quite similar to the original aside from the height of the enclosure. However, the funnel was the same, which could explain why this design performed better than the other new prototypes in low mosquito density and similarly in high density. The circular funnel of prototypes 3 and 4 may explain why they did not catch as many mosquitoes. Moreover, the smaller, horizontal enclosure screen and reduced airflow in prototypes 3 and 4 may contribute to the lower number of mosquitoes compared to the original (P1) and medium (P2). Several studies have demonstrated that some variables such as vertical or horizontal screens, air flow and direction, trap colour, screen mesh size, etc. could affect the trap’s effectiveness [25, 26]. The minimal expected trapping rate that is required for such a trap to be effective to protect the inhabitants is a 70% density reduction from what is expected with other malaria control means [27].

Since the establishment of a link between mosquitoes and malaria transmission [11], house screening was one of the first experiments used as part of malaria vector management [28]. Recent studies using house screening have found a reduced number of mosquitoes entering the house and the consequent protection of households against mosquito bites compared to houses without a screen [19, 29, 30]. In addition to house screening, other studies demonstrate that houses with fewer openings for mosquitoes to enter through can help reduce malaria transmission by lowering human exposure to infectious bites [31, 32]. Traps could have an additional, confounding effect when used with long-lasting insecticidal nets (LLINs) or Indoor Residual Spray (IRS), further decreasing the number of malaria vectors entering homes and biting occupants. Furthermore, trap efficiency in houses without suitable openings could be trapping mosquitoes that may escape any other malaria control measures in place (e.g. LLINs), and as a consequence, such traps could further protect the overall population in the village.

As such, the use of LFETs could be an effective and relatively simple method of reducing indoor mosquito vector densities generally in a local area, and consequently this could decrease malaria transmission and avert the harmful effects of other insects. This would be especially powerful in communities where all households commit to the LFET’s proper use. This is in contrast to window screening alone, as in this case mosquitoes are blocked from accessing a given house but are not trapped and so are free to continue to try to find a suitable place to feed, transmit pathogens, and reproduce. Traps do not discriminate by age or insecticide susceptibility, which is an issue with the insecticide-based malaria control tools. For instance, Vallée du Kou is an irrigated area where pesticides are extensively used in rice and cotton fields surrounding the village, and a significant proportion of mosquitoes exhibited high resistance level to pyrethroids [19, 21, 33].

Malaria control is undermined by the growing proportion of outdoor feeding mosquitoes [9–11] due to the use of insecticide in LLINs and IRS. The LFET helps remove some outdoor fed mosquitoes looking for resting areas. In this study, *An. gambiae* was the main species collected in both sites. Female *Anopheles* mosquitoes regularly take blood meal every three days, and these are mostly in human dwellings [6–8]. During this study, the LFETs protected people from 43% of the blood seeking *An. gambiae* female mosquitoes.

The LFET is simple to produce locally, it contains no chemicals or attractants, it is easy to install, and it operates with no additional user work. The medium trap prototype is small enough to favour its use by owners while keeping its killing effect on mosquitoes. Considering the current cost, the LFET prototypes manufactured locally using other materials such as aluminium or recycled plastic, can reduce their mass-production for use at a village scale. Additionally, a long-term use of the LFET prototypes as malaria control tool requires low maintenance but cleaning. However, the frequency of cleaning would depend on the density of mosquitoes trapped over a given period. For instance, in endemic areas with high mosquito density, a monthly cleaning-period would be enough to deny attracting mosquito predators, which could damage the nettings. In low mosquito density area, the cleaning frequency would be performed according to the owner convenience. For the trap cleaning, the down sleeve could be untied and used by opening slowly to collect the dead mosquitoes while avoiding live mosquitoes’ escapes.

During this LFET study, some parameters such as humidity, rainfall, temperature could not be assessed in the village, which could have enabled us to check the environment’s effects on trap efficiency. These aspects are considered as limitations of this study and could be included in future studies.

## Conclusion

When tested in both high and low mosquito density settings, the medium (P2) LFET design showed a promising level of performance in reducing mosquito numbers within dwellings. This suggests it may be used as a vector control tool to further suppress malaria mosquito populations. In addition, the trap protects residents from other harmful insects, such as vectors of arboviruses.

Although these new, smaller LFET prototypes have been developed and shown promise, further studies at a village scale will be needed to assess community acceptance of the traps, as well as their ability to control malaria vector populations.

## Supplementary Information

**Additional file 1: Fig. 10**. (a) Number of mosquitoes collected per trap versus house in VK3 and (b) in Soumousso over the study period.

## Data Availability

All data generated and analysed during this study are available on Github: https://github.com/RogerSANOU/Lehmann-trap-dataset.git.
