# Supplementary material for: Assessment of novel Lehmann’s funnel entry trap prototypes performance to control malaria mosquito populations
Source: Malar J. 2021 Jan 1;20:2. doi: 10.1186/s12936-020-03532-x (PMC7777431; doi:10.1186/s12936-020-03532-x)
Supplement: Supplementary file 1 — Additional file 1: Fig. 10. (a) Number of mosquitoes collected per trap versus house in VK3 and (b) in Soumousso over the study period. [file 12936_2020_3532_MOESM1_ESM.pptx]

## Slide 1
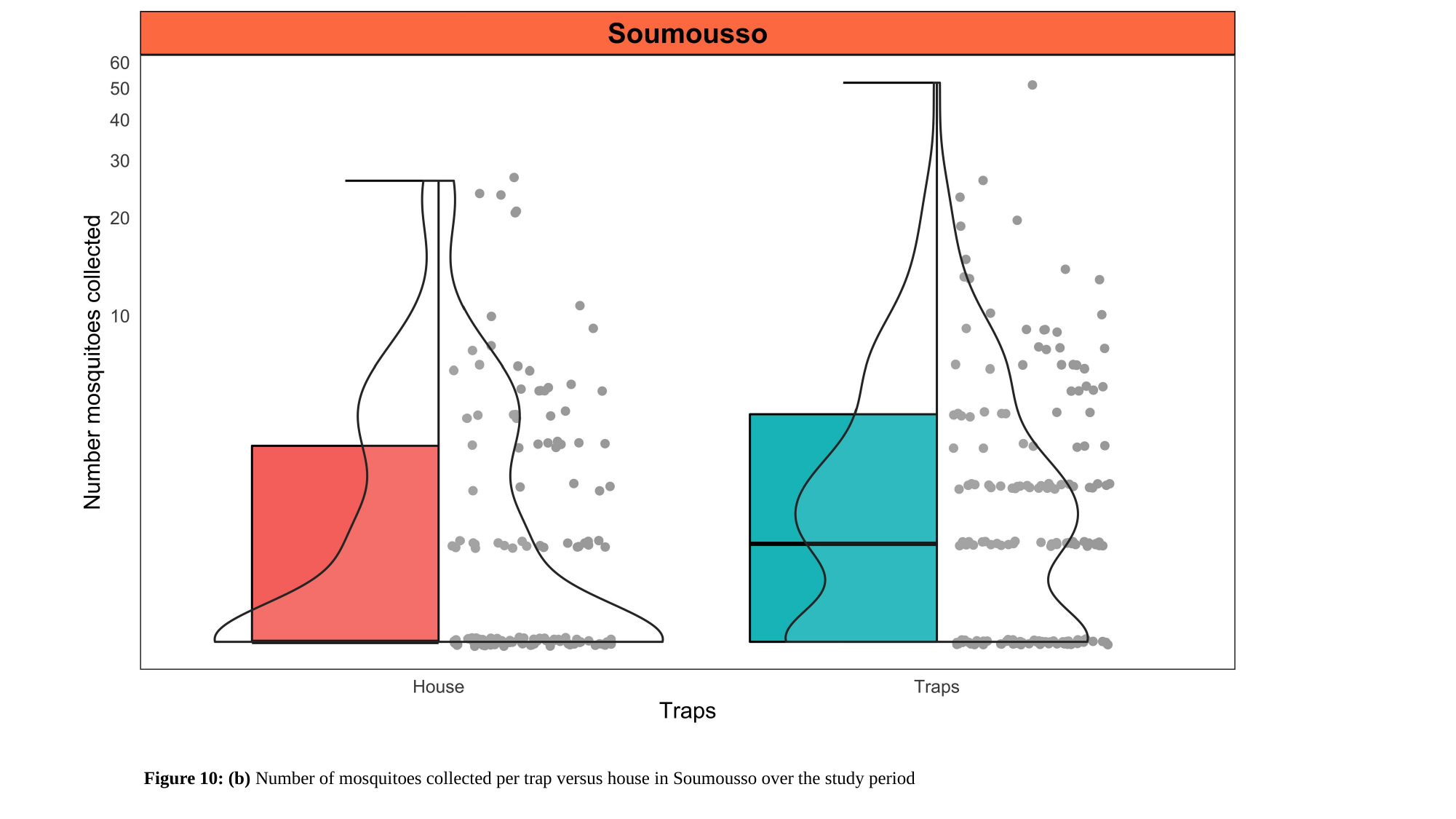

Figure 10: (b) Number of mosquitoes collected per trap versus house in Soumousso over the study period

## Slide 2
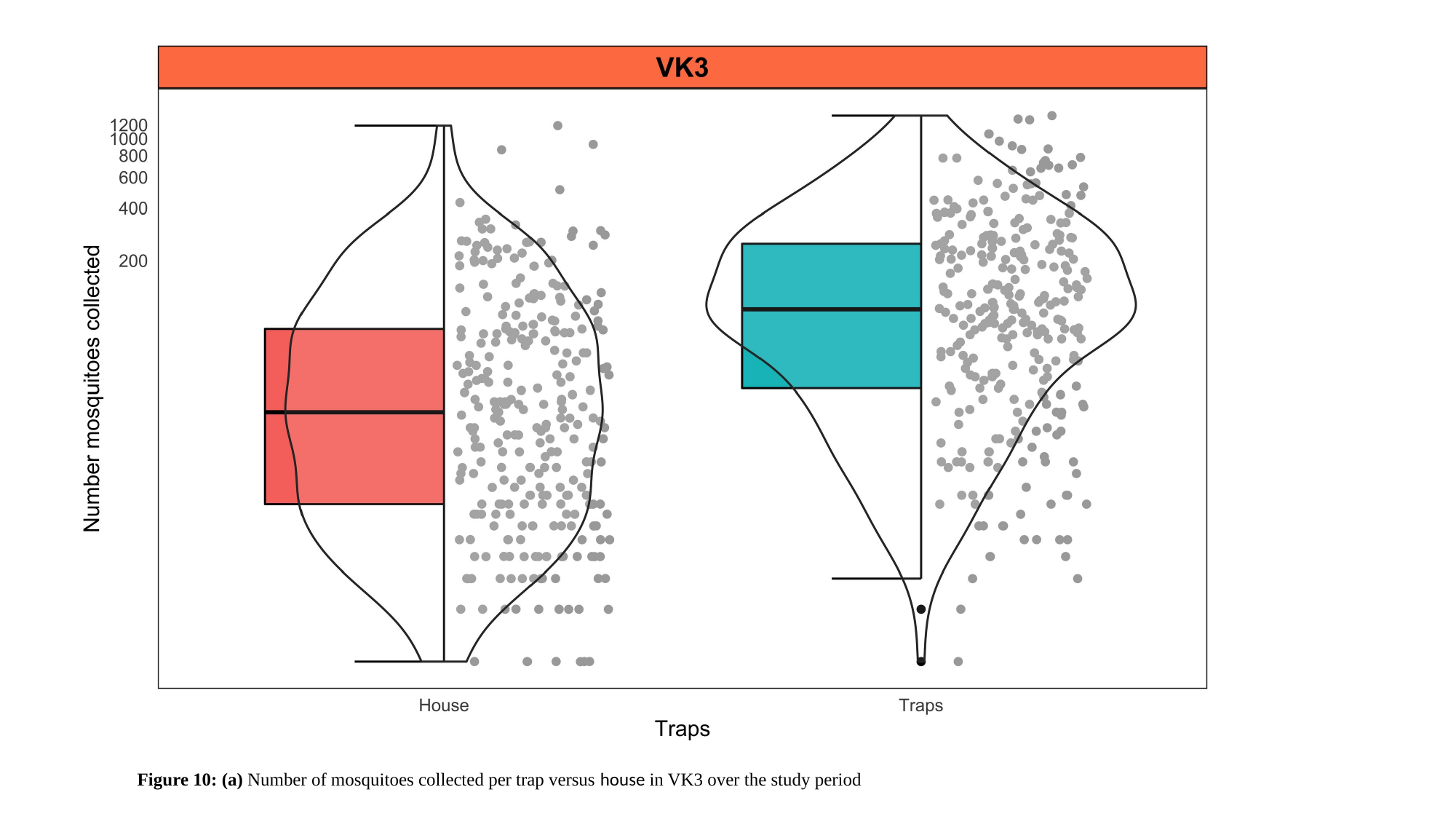

Figure 10: (a) Number of mosquitoes collected per trap versus house in VK3 over the study period
